# Supplementary material for: Regulation of replication origin licensing by ORC phosphorylation reveals a two-step mechanism for Mcm2-7 ring closing
Source: Proc Natl Acad Sci U S A. 2023 Jul 10;120(29):e2221484120. doi: 10.1073/pnas.2221484120 (PMC10629557; doi:10.1073/pnas.2221484120)
Supplement: Supplementary file 1 — Appendix 01 (PDF) [file pnas.2221484120.sapp.pdf]

PNAS

Supporting Information for

Regulation of replication origin licensing by ORC phosphorylation reveals a two-step mechanism for Mcm2-7 ring closing

Audra L. Amasino[1], Shalini Gupta[1], Larry J. Friedman[2], Jeff Gelles[2]*, and Stephen P Bell[1]*

*Co-corresponding authors:
Stephen P. Bell
Email : spbell@mit.edu
Phone : 617-253-2054

Jeff Gelles
Email : gelles@brandeis.edu
Phone : 781-736-2377

This PDF file includes:

Figures S1 to S8
Tables S1 to S3

Other supporting materials for this manuscript include the following:

Datasets and Matlab analysis programs stored in Zenodo.

Supplementary figures

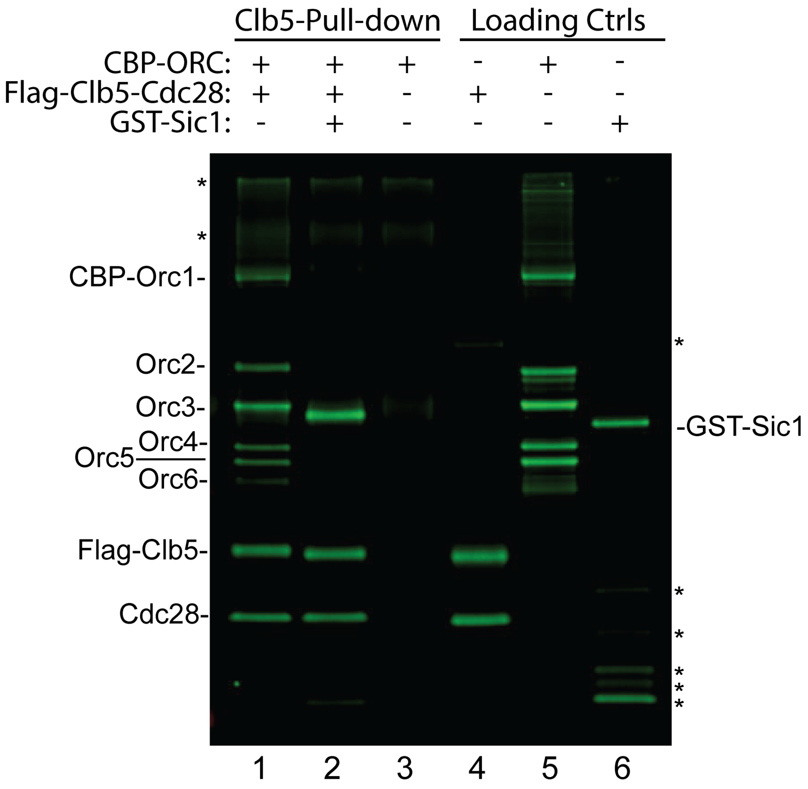

Supp. Fig. 1. Sic1 disrupts binding of Clb5-CDK to ORC.

Flag-coprecipitation experiments were performed with the indicated proteins. When present, GST-Sic1 was added for 5 min. after the initial incubation. Bead associated proteins were separated by SDS-PAGE. Purified protein loading controls were: Flag-Clb5-Cdc28 (100% of input, lane 4); ORC (2.5% of input, lane 5); and GST-Sic1 (30% of input, lane 6). Asterisks denote either contaminants or degradation products.

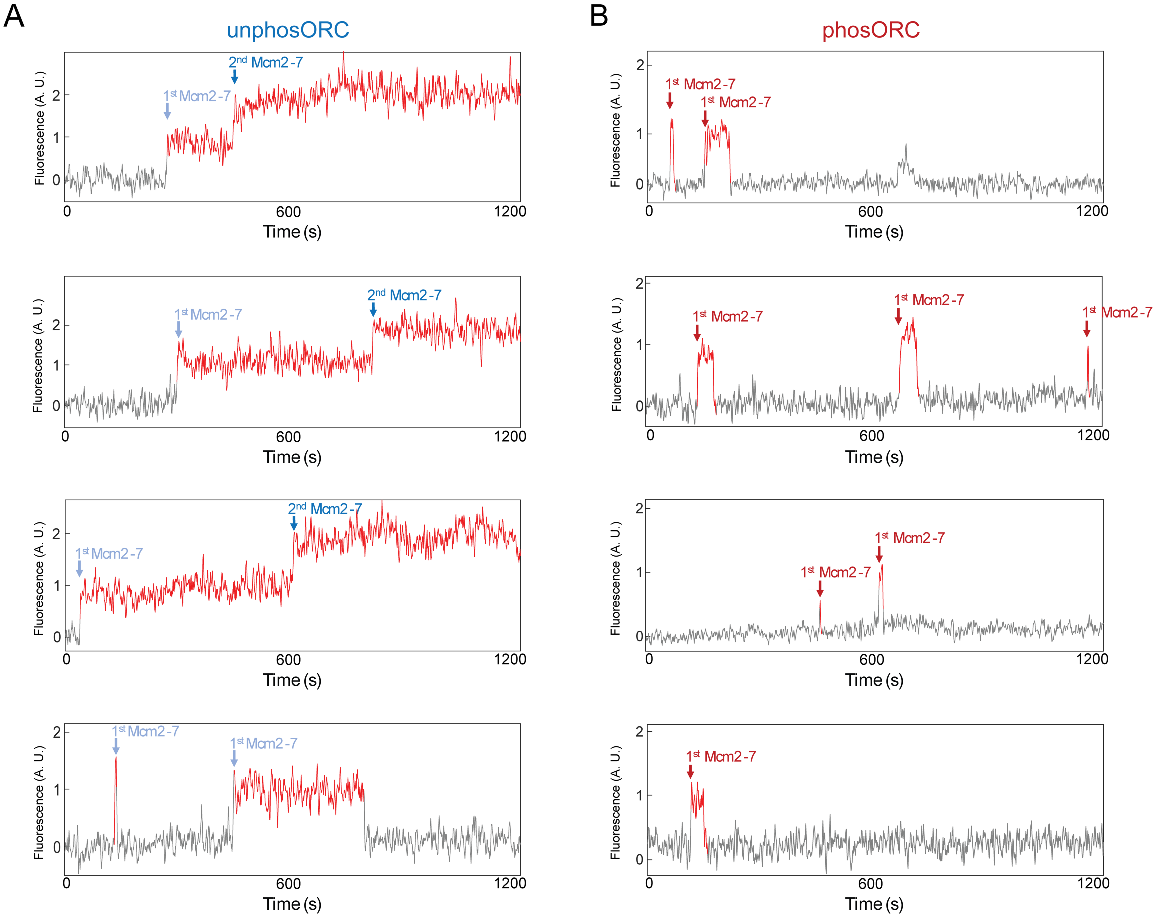

Supp. Fig. 2. Additional fluorescence records showing Mcm2-7$^{4SNAP\text{-}JF646}$ association with single DNA molecules.

A. Additional example records of Mcm2-7$^{4SNAP\text{-}JF646}$ associations with an individual DNA molecule in the presence of unphosORC (experiment same as in Fig. 1), plotted as in Fig. 2A.
B. Additional example records of Mcm2-7$^{4SNAP\text{-}JF646}$ associations with an individual DNA molecule in the presence of phosORC (experiment same as in Fig. 1), plotted as in Fig. 2A.

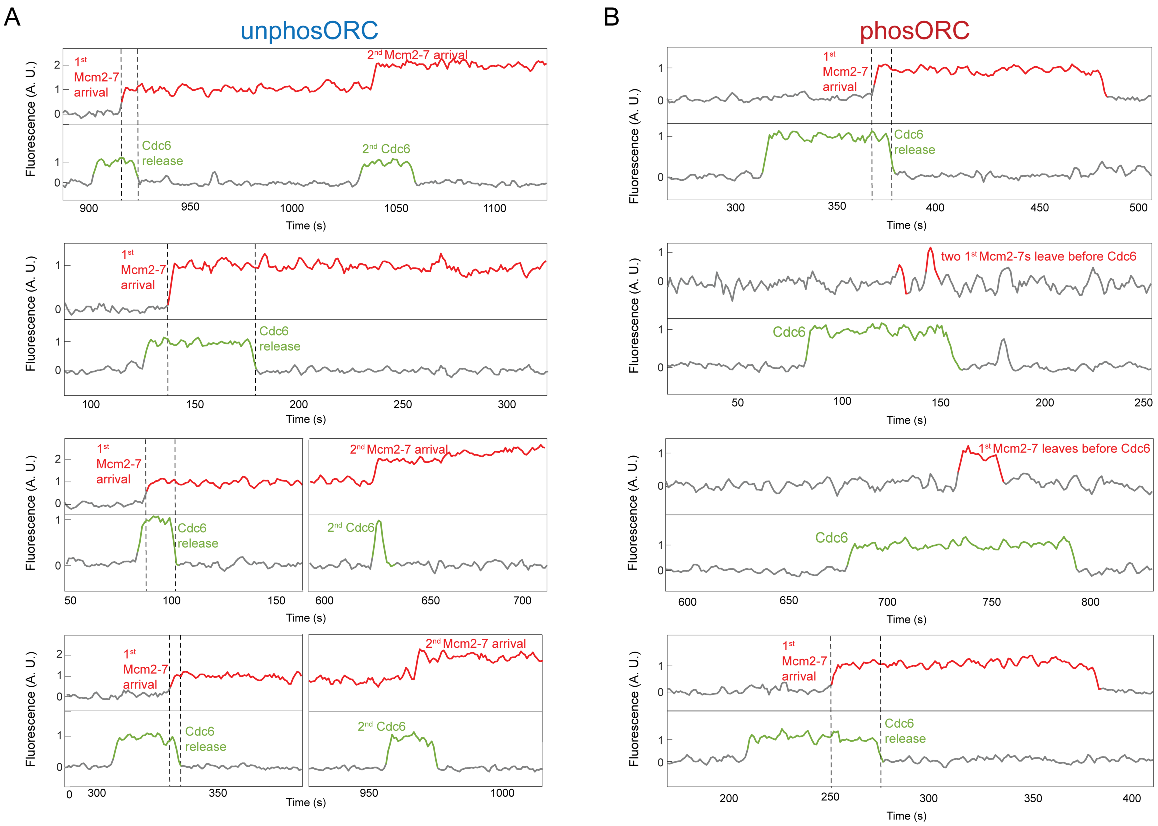

Supp. Fig. 3. Additional fluorescence records of Mcm2-7 and Cdc6 interactions with single DNA molecules.

A. Additional example records of Mcm2-7$^{4SNAP-DY649}$ (red) and Cdc6$^{SORT-DY549}$ (green) records from a single-molecule helicase-loading experiment with unlabeled Cdt1 and unphosORC. Dashed lines and plotting are as described in Fig. 4B.

B. Additional Mcm2-7$^{4SNAP-DY649}$ (red) and Cdc6$^{SORT-DY549}$ (green) records from a single-molecule helicase-loading experiment with phosORC. Dashed lines and plotting are as described in Fig. 4B.

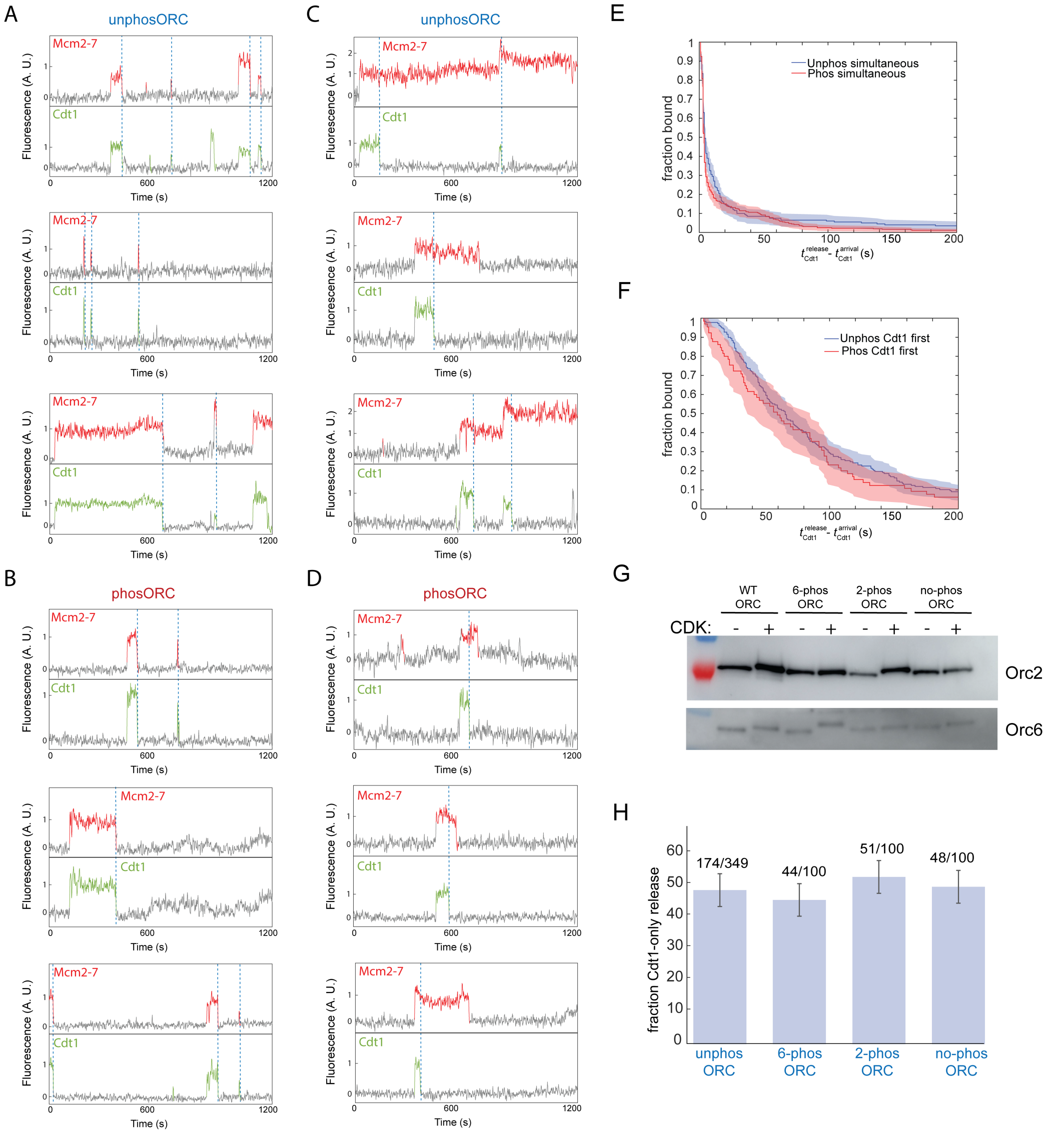

Supp. Fig 4. Additional Mcm2-7/Cdt1 fluorescence intensity traces, Cdt1 dwell-time distributions, and non-CDK-treated ORC phospho-site mutant controls.

A,B. Additional example records showing non-productive Mcm2-7$^{4SORT\text{-}JF646}$ (red) and Cdt1$^{SORT\text{-}DY549}$ (green) simultaneous dissociation (dashed lines) from a single DNA (left pathway in Fig. 5A) for unphosORC (A) or phosORC (B). Plotted as in Fig. 5B.

C,D. Additional example records showing potentially productive Mcm2-7$^{4SORT\text{-}JF646}$ (red) and Cdt1$^{SORT\text{-}DY549}$ (green) in which Cdt1 dissociates from DNA before the corresponding first Mcm2-7 (dashed lines; right pathway in Fig. 5A) in the presence of unphosORC (C) or phosORC (D). Plotted as in Fig. 5C.

E. The fraction of Cdt1 molecules that remain on DNA for events where Mcm2-7 and Cdt1 leave simultaneously in the presence of unphosORC (blue), or phosORC, (red) measured from the time of Cdt1 arrival. Shaded areas represent 95% confidence intervals.

F. The fraction of Cdt1 molecules that remain on DNA for events where Cdt1 leaves before the associated Mcm2-7 in the presence of unphosORC (blue), or phosORC, (red) measured from the time of Cdt1 arrival. Shaded areas represent 95% confidence intervals.

G. Western blot of Orc2 and Orc6 showing CDK-dependent band-size increases for wt, 6-phos, 2-phos, and no-phos ORC. WT-ORC shows a CDK-dependent shift in both Orc2 and Orc6, whereas the 6-phos, 2-phos, and no-phos mutants show only shifts in Orc6, Orc2, or no band shifts, respectively.

H. The percentage (± SEM) of first Cdt1 dissociation events that followed the productive pathway (Cdt1 release without Mcm2-7 release) by unphosORC and the unphosphorylated (non-CDK-treated) ORC phospho-site mutants.

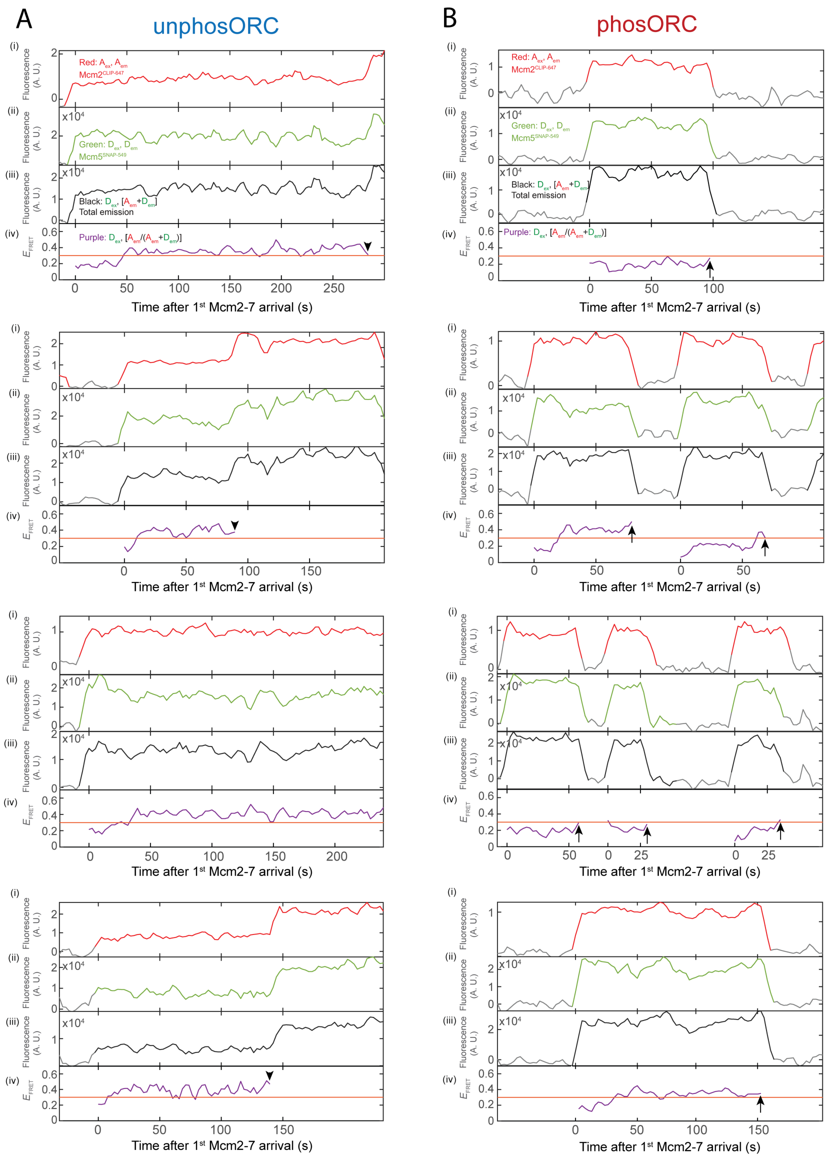

Supp. Fig. 5. Additional Mcm2-7^{25FRET} ring-closing FRET records from individual DNA molecules.

A. Additional example records of Mcm2-7^{25FRET} association with a single DNA molecule in the presence of unphosORC. Panels are as described in Fig. 6B. E_{FRET} is shown only during intervals when fluorescence from both labeled subunits was present, or until a 2nd Mcm2-7 arrived (marked with arrowhead).

B. Additional example records of Mcm2-7^{25FRET} association with a single DNA molecule in the presence of phosORC. Panels are as described in Fig. 6B. 1st Mcm2-7 release events are marked with arrow.

A B

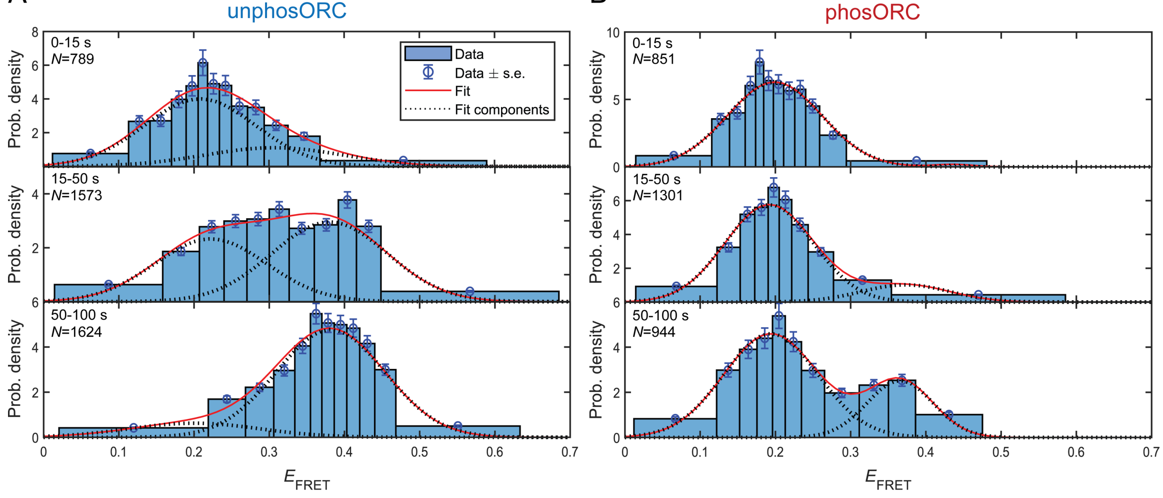

C

Time interval (s)	Parameter	Value ± S.E.
0-15	P_{low}	0.7 ± 0.6
	μ_{low}	0.21 ± 0.02
	σ_{low}	0.07 ± 0.01
	μ_{high}	0.31 ± 0.02
	σ_{high}	0.09 ± 0.05
15-50	P_{low}	0.4 ± 0.1
	μ_{low}	0.22 ± 0.02
	σ_{low}	0.07 ± 0.01
	μ_{high}	0.38 ± 0.02
	σ_{high}	0.08 ± 0.01
50-100	P_{low}	0.14 ± 0.09
	μ_{low}	0.20 ± 0.06
	σ_{low}	0.09 ± 0.02
	μ_{high}	0.38 ± 0.01
	σ_{high}	0.07 ± 0.01

D

Time interval (s)	Parameter	Value ± S.E.
0-15	P_{low}	0.99 ± 0.01
	μ_{low}	0.20 ± 0.004
	σ_{low}	0.06 ± 0.003
	μ_{high}	0.43 ± 0.02
	σ_{high}	0.03 ± 0.02
15-50	P_{low}	0.85 ± 0.03
	μ_{low}	0.19 ± 0.004
	σ_{low}	0.06 ± 0.003
	μ_{high}	0.37 ± 0.02
	σ_{high}	0.06 ± 0.01
50-100	P_{low}	0.72 ± 0.04
	μ_{low}	0.19 ± 0.01
	σ_{low}	0.06 ± 0.01
	μ_{high}	0.37 ± 0.01
	σ_{high}	0.04 ± 0.01

Supp. Fig. 6. Distributions of Mcm2-7^{25FRET} ring-closing E_{FRET} values.

A. Probability-density histograms of unphosORC-directed Mcm2-7^{25FRET} E_{FRET} values (excluding outliers < 0.1 or > 0.7, 0.7% of data) for the indicated time intervals after 1st Mcm2-7 arrival. Each histogram was fit with two-component Gaussian mixture models (lines). Note: *N* values represent number of data points within each time-slice, not the number of Mcm2-7s (this number is given in Fig. 6C).

B. Probability-density histograms of phosORC-directed Mcm2-7^{25FRET} E_{FRET} values (excluding outliers < 0.1 or > 0.7, 1.8% of data) for the indicated time intervals after 1st Mcm2-7 arrival. Each histogram was fit with a two-component Gaussian mixture models (lines).

C. Fit parameters for the unphosORC-directed Mcm2-7^{25FRET} E_{FRET} values. Values are reported for each time interval and were determined separately for each distribution.

D. Fit parameters for phosORC-directed Mcm2-7^{25FRET} E_{FRET} values. Values are reported for each time interval and were determined separately for each distribution.

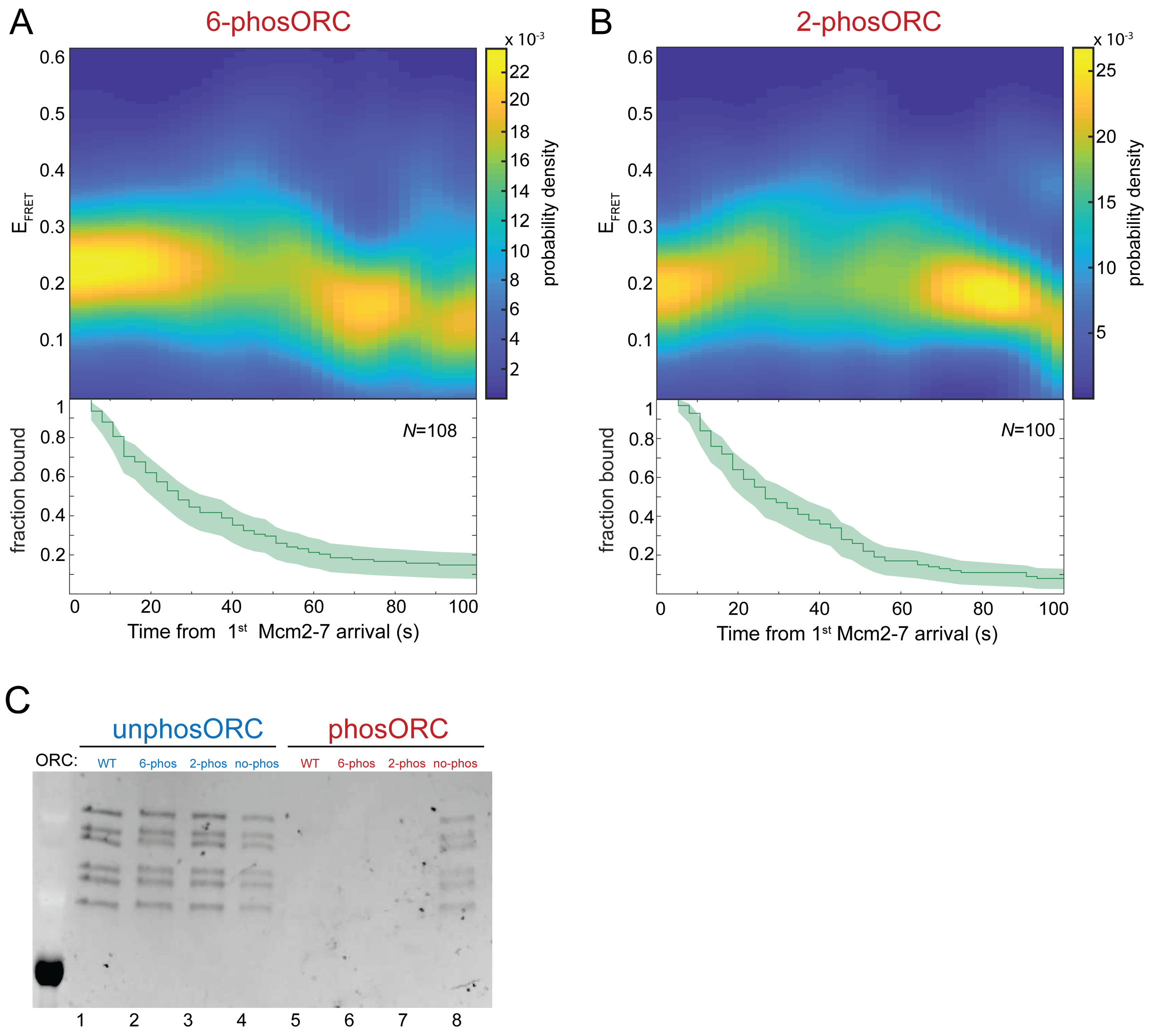

Supp. Fig. 7. ORC with only the ORC6 subunit phosphorylated or only the ORC2 subunit phosphorylated inhibits Mcm2-7 ring closing and loading.

A. Heat map of E_{FRET} values vs. time after first Mcm2-7^{25FRET} binding for N = 108 DNA-bound 1st Mcm2-7 molecules in a reaction using 6-phosORC (top). Data were selected and plotted as described in Fig. 6C. The fraction of such complexes remaining at each time point is plotted (bottom).

B. Heat map of E_{FRET} values vs. time after first Mcm2-7^{25FRET} binding for N = 100 DNA-bound 1st Mcm2-7 molecules in a reaction using 2-phosORC (top). The data were selected and plotted as described in Fig. 6C. The fraction of such complexes remaining at each time point is plotted (bottom).

C. Bulk helicase-loading assays comparing the loading of WT ORC (lanes 1 and 5), 2-phosORC (lanes 2 and 6), 6-phosORC (lanes 3 and 7) and no-phosORC (lanes 4 and 8) with and without phosphorylation. A high-salt wash (500 mM NaCl) was used to remove all helicase-loading intermediates from DNA.

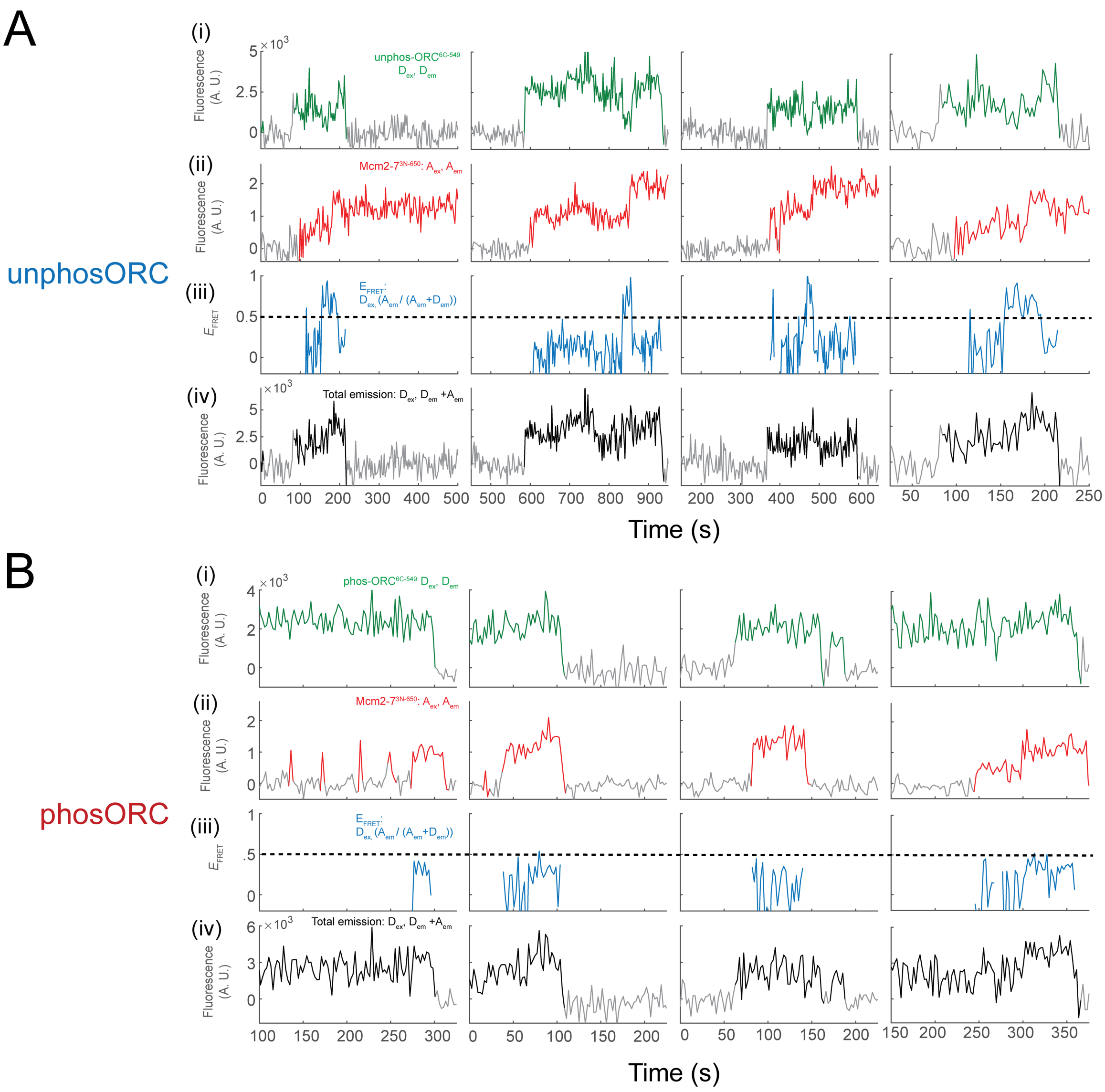

Supp. Fig. 8. Additional records from individual DNA molecules for single-molecule MO-complex formation FRET assay.

A. Additional example records of MO formation assays monitoring unphosORC$^{6C\text{-}DY549}$ and Mcm2-7$^{3N\text{-}DY650}$ association with a single DNA molecule. Panels are as described in Fig. 7B.

B. Additional example records of MO formation assays monitoring phosORC$^{6C\text{-}DY549}$ and Mcm2-7$^{3N\text{-}DY650}$ association with a single DNA molecule. Panels are as described in Fig. 7B.

Table S1: Fit parameters for Mcm2-7 binding to ORC-Cdc6-DNA.

ORC state	A_f	k_a (× 10^{-3} s^{-1})	$k_{n,s}$ (× 10^{-3} s^{-1})
unphosORC	0.82 ± 0.02	4.7 ± 0.3 (N=466 DNA)	0.09 ± 0.01 (N=625 non-DNA locations)
phosORC	0.68 ± 0.03	2.0 ± 0.2 (N=612 DNA)	0.09 ± 0.01 (N=526 non-DNA locations)

Fits are for a single exponential binding model adjusted to remove the contribution from non-specific background DNA binding. Kinetic model is described in the Methods.
k_a is the apparent first-order rate constant for Mcm2-7-Cdt1 binding to DNA. $k_{n,s}$ is the apparent first order rate constant for Mcm2-7-Cdt1 binding to control non-DNA sites (ie. non-specific binding to the surface of the slide). A_f represents the "active fraction" of DNA molecules capable of recruiting Mcm2-7-Cdt1.

Table S2. Yeast strains used in this study.

Strain name	Expresses	Strain details	Study
ySDORC	Wild-type ORC	*MATa ade2-1 ura3-1 his3-11,15 trp1-1 leu2-3,112 can1-100<br>bar1::hyg pep4::kanMX<br>TRP1::pJF18 (GAL1,10 ORC5-opt ORC6-opt) HIS3::pJF17 (GAL1,10 ORC3-opt ORC4-opt) URA3::pJF19 (GAL1,10 CBP-TEV ORC1-opt ORC2-opt)*	Frigola et al., 2013
yAA01	6-phosORC	*MATa ade2-1 ura3-1 his3-11,15 trp1-1 leu2-3,112 can1-100<br>bar1::hyg pep4::kanMX<br>TRP::pJF18 (GAL1,10 ORC5-opt ORC6-opt) HIS3::pJF17 (GAL1,10 ORC3 ORC4)<br>URA3::pAA04 (GAL1,10 Ubi-GGG-3x-Flag-ORC1 +ORC2-6Ala) ORC2-V5 (NatMX)*	This study
yAA02	2-phosORC	*MATa ade2-1 ura3-1 his3-11,15 trp1-1 leu2-3,112 can1-100<br>bar1::hyg pep4::kanMX<br>TRP1::pAA02 (GAL1,10 ORC5-opt ORC6-opt-4Ala) HIS3::pJF17 (GAL1,10 ORC3-opt ORC4-opt) URA3::pAA04 (GAL1,10 Ubi-GGG-3x-Flag-ORC1-opt ORC2-opt)<br>ORC6-V5 (HphMX)*	This study
yAA03	no-phosORC	*MATa ade2-1 ura3-1 his3-11,15 trp1-1 leu2-3,112 can1-100<br>bar1::hyg pep4::kanMX<br>TRP1::pAA02 (GAL1,10 ORC5-opt ORC6-opt-4Ala) HIS3::pJF17 (GAL1,10 ORC3-opt ORC4-opt) URA3::pAA04 (GAL1,10 Ubi-GGG-3x-Flag-ORC1-opt ORC2-opt-6Ala<br>ORC6-V5 (HphMX)*	This study
yST166	MCM2-7^{4SNAP} and $Cdt1^{Sort}$	*ade2-1 trp1-1 leu2-3,112 his3-11,15 ura3-1 can1-100 bar1::HisG lys2::HisG pep4::unmarked HIS3::pSKM004 (GAL1,10-MCM2, Flag-MCM3) URA3::pALS3 (GAL1,10 UbSORT-Cdt1, GAL4) LYS2::pST022 (GAL1,10 SNAP-MCM4, MCM5) TRP1::pSKM003 (GAL1,10 MCM6, MCM7)*	Ticau et al., 2015

yST180	Mcm2-7^{4Sort} and Cdt1	*ade2-1 trp1-1 leu2-3,112 his3-11,15 ura3-1 can1-100 bar1::HisG lys2::HisG pep4::unmarked TRP1::pSKM003 (GAL1,10-MCM6,MCM7) URA3::pALS1(GAL1,10-Cdt1,GAL4) HIS3::pSKM004(GAL1,10-MCM2,Flag-MCM3) LYS2::pST034 (GAL1,10 UbiSORT-MCM4,MCM5)*	Gupta et al., 2021
yST229	Mcm2-7^{25FRET}	*ade2-1 trp1-1 leu2-3,112 his3-11,15 ura3-1 can1-100 bar1::HisG lys2::HisG pep4::unmarked TRP1::pSKM003 (GAL1,10-MCM6,MCM7) URA3::pALS1(GAL1,10-Cdt1,GAL4) HIS3::pST058 (GAL1,10 MCM2-721-CLIP,Flag-MCM3) LYS2::pST057 (GAL1,10 MCM4, MCM5-591-SNAP)*	Ticau et al., 2017
yST103	Cdt1^{Sort}	*ade2-1 trp1-1 leu2-3,112 his3-11,15 ura3-1 can1-100 bar1::HisG lys2::HisG pep4::unmarked URA3::pST013 (GAL1,10 UbSORT-Cdt1-Flag)*	Gupta et al., 2021
ySG39	ORC^{6C-549}	*ade2-1 trp1-1 leu2-3,112 his3-11,15 ura3-1 can1-100 bar1::HisG lys2::HisG pep4::unmarked LYS2::pSKM002 (GAL1,10 MCM4, MCM5) TRP1::pSKM003 (GAL1,10 MCM6, MCM7) HIS3::pSG13 (GAL1, 10 MCM2, Flag-TEV-GG-MCM3)*	Gupta et al., 2021
ySG24	Mcm2-7^{3N-650}	*ade2-1 trp1-1 leu2-3,112 his3-11,15 ura3-1 can1-100 bar1::hisG lys2::HisG pep4::unmarked LYS2::pSKM002 (GAL1,10 MCM4, MCM5) TRP1::pSKM003 (GAL1,10 MCM6, MCM7) HIS3::pSG13 (GAL1,10 MCM2, Flag-TEV-GG-MCM3)*	Gupta et al., 2021
ySG60	6-phos ORC^{6C-549}	*MATa ade2-1 ura3-1 his3-11,15 trp1-1 leu2-3,112 can1-100 bar1::hyg pep4::kanMX HI3::pJF17 (GAL1,10 ORC3-opt, ORC4-opt) TRP1::pAZ63 (Gal1,10 ORC5-opt, ORC6-opt-C-LPETGG) URA3::pSG52 (Gal1,10 CBP-ORC1-opt, ORC2-opt-6A)*	This study
ySG61	2-phos ORC^{6C-549}	*MATa ade2-1 ura3-1 his3-11,15 trp1-1 leu2-3,112 can1-100 bar1::hyg pep4::kanMX*	This study

		HIS3::pJF17 (GAL1,10 ORC3-opt, ORC4-opt) TRP1::pSG53 (GAL1,10 ORC5-opt, ORC6-opt-4A-C-LPETGG) URA3::pJF19(GAL1,10 CBP-ORC1-opt, ORC2-opt) ORC6-V5 (HphMX)	
ySK119	Clb5-Cdk1	*ade2-1 trp1-1 leu2-3,112 his3-11,15 ura3-1 can1-100 bar1::HisG lys2::HisG pep4::unmarked URA3::GAL1,10 Δ2-95-CLB5-Flag CDC28-His*	Looke et al., 2017

Table S3. Plasmids used in this study

Plasmid name	Construct encodes	Description	source
pSKM033	Flag-Cdc6	pGEX- GST-PP-FLAG-Cdc6	Kang et al., 2014
pET-GSS-Cdc6	Cdc6-sort	pET23b-GST-SUMO-GGG-Cdc6	Ticau et al., 2015
pAA02	Orc5 and Orc6-4Ala	*GAL1,10 ORC5-opt*, *ORC6-opt-4Ala*	This study
pAA04	UbSORT-FLAG-Orc1 and Orc2-6Ala	*GAL1,10 UBI-GGG-3xFlag-ORC1-opt, ORC2-opt-6Ala*	This study
pJF17	Orc3 and Orc4	*GAL1-,10 ORC3-opt, ORC4-opt*	Frigola et al., 2013
pJF18	Orc5 and Orc6	*GAL1-,10 ORC5-opt, ORC6-opt*	Frigola et al., 2013
pJF19	CBP-Orc1 and Orc2	*GAL1,10 ORC1-opt ORC2-opt*	Frigola et al., 2013
pSG52	CBP-Orc1 and Orc2-6Ala	*GAL1,10 CBP-ORC1-opt, ORC2-opt-6Ala*	This study
pSG53	Orc5 and Orc6-4Ala-CLPETGG	*GAL1,10 ORC5-opt, ORC6-opt-4A-C-LPETGG*	This study
pAZ63			
pSG13	Mcm2 and Flag-TEV-GG-Mcm3	*GAL1,10 MCM2, Flag-TEV-GG-MCM3*	Gupta et al., 2021
pSKM002	Mcm4 and Mcm5	*GAL1,10 MCM4, MCM5*	Kang et al., 2014
pSKM003	Mcm6 and Mcm7	*GAL1,10 MCM6, MCM7*	Kang et al., 2014
pSKM004	Mcm2 and Flag-Mcm3)	*GAL1,10 MCM2, Flag-MCM3*	Kang et al., 2014
pST022	SNAP-Mcm4 and Mcm5	GAL1,10 SNAP-MCM4, MCM5	Ticau et al., 2015
pALS1	Cdt1 and Gal4	*GAL1,10 CDT1, GAL4*	Kang et al., 2014
pALS3	UbSORT-Cdt1 and Gal4	*GAL1,10 UbSORT-CDT1, GAL4*	Ticau et al., 2015

pST034	UbiSORT-Mcm4 and Mcm5	*pST034 GAL1,10 UbiSORT-MCM4, MCM5*	Ticau et al., 2015
pST013	UbSORT-Cdt1-Flag	*GAL1,10-UbSORT-CDT1-Flag*	Gupta et al., 2021
pST058	Mcm2-721-CLIP and Flag-Mcm3	*GAL1,10 MCM2-721-CLIP, Flag-MCM3*	Ticau et al., 2017
pST057	Mcm4 and Mcm5-591-SNAP	*GAL1,10 MCM4, MCM5-591-SNAP*	Ticau et al., 2017
pGEX-Sic1	GST-Sic1	*pGEX-4T-2-GST-Sic1*	Heller et al. 2011
